# Uncovered variability in olive moth (*Prays oleae*) questions species monophyly

**DOI:** 10.1371/journal.pone.0207716

**Published:** 2018-11-26

**Authors:** Tânia Nobre, Luis Gomes, Fernando Trindade Rei

**Affiliations:** Laboratory of Entomology, ICAAM—Instituto de Ciências Agrárias e Ambientais Mediterrânicas, Universidade de Évora, Évora, Portugal; Sichuan University, CHINA

## Abstract

The olive moth -*Prays oleae* Bern.- remains a significant pest of olive trees showing situation dependent changes in population densities and in severity of damages. The genetic variability of olive moth was assessed on three main olive orchards regions in Portugal by three different markers (*COI*, *nad5* and *RpS5*), suggesting high species diversity albeit with no obvious relation with a regional pattern nor to an identified ecological niche. Selected *COI* sequences obtained in this study were combined with those available in the databases for *Prays* genus to generate a global dataset. The reconstruction of the *Prays* phylogeny based on this marker revealed the need to revise *Prays oleae* to confirm its status of single species: *COI* data suggests the co-existence of two sympatric evolutionary lineages of morphologically cryptic olive moth. We show, however, that the distinct mitochondrial subdivision observed in the partial COI gene fragment is not corroborated by the other DNA sequences. There is the need of understanding this paradigm and the extent of *Prays* variability, as the disclosure of lineage-specific differences in biological traits between the identified lineages is fundamental for the development of appropriate pest management practices.

## Introduction

Olive is an ancient ubiquitous crop of considerable socioeconomic importance, being a major agro-ecosystem in the Mediterranean basin. For the Mediterranean region, three main olive pests have been recognized: the olive fruit fly, *Bactrocera oleae* Gmelin, the olive moth, *Prays oleae* Bern. and the black scale, *Saissetia oleae* Bern. [1–3]. The importance of the last two has decreased as a whole due to advances in olive pest management [3], but regional relevance persists.

The olive moth, *Prays oleae* (Lepidoptera, Yponomeutidae) remains an abundant pest of olive trees throughout the Mediterranean and the Black Sea, the Middle East and Canary Islands [4]. Undertaking three generations per year, and with the larval stages attacking different organs of the tree, its’ action can increase fruit fall and damage leaves, flowers and fruits. The olive moth is thus being held responsible for high losses in the olive yield [5], lowering tree growth, fruit set and fruit/oil quality. In north Portugal this moth competes in importance with the olive fruit fly, being considered the most important olive tree pests due to the large production losses [6]. There is empirical indication that the seriousness of the losses due to *Prays oleae* are highly variable, depending both on time (crop seasons) and space (regions).

The degree of synchrony between adult emergence and the olive fruit suitability for oviposition by egg-laying moth females varies greatly from year to year [7]. This synchrony can account for part of the seasonal variability of the losses caused by the olive moth. Predators (like ants, chrysopids, anthocorids and spiders; e.g.[8]) and parasitoids (mainly egg parasitoid *Trichogramma* species, but others have also been referenced, e.g. [9–11]) are also likely responsible for the observed variability, both seasonal and regional. The state and composition of the functional diversity associated to the olive grove is highly affected by crop management practices (including tillage and the use of pesticides) impacting also at a landscape/regional scale (e.g. [12,13]).

Agricultural systems, including olive groves, form a mosaic at a landscape scale shifting both in time and space, that might induce differentiation and determine the population structure of the *Prays oleae* (as it depends on olive trees for survival). Because genetic variation is essential for the adaptability of a population, the selection of fitness‐related traits might be driving changes in population densities and severity of the pest.

In this study we look into the genetic variability of *Prays oleae* on three main olive grove regions in Portugal by means of sequencing two selected mitochondrial DNA amplicons and a nuclear DNA amplicon. To the best of our knowledge, this is the first systematic study looking into the population(s) genetic variability of *Prays oleae*. The reconstruction of the *Prays* phylogeny based on *COI* revealed the need to revise *Prays oleae* to confirm its status of single species and assess its relation with other *Prays* species, in particular with *Prays fraxinella*. Furthermore, the phylogenetic and the network analyses of the variability here performed suggest cryptic species diversity of the olive moth, albeit not clearly linked to regional patterns.

## Methodology

### Taxon sampling and data collection

**Fig 1:**
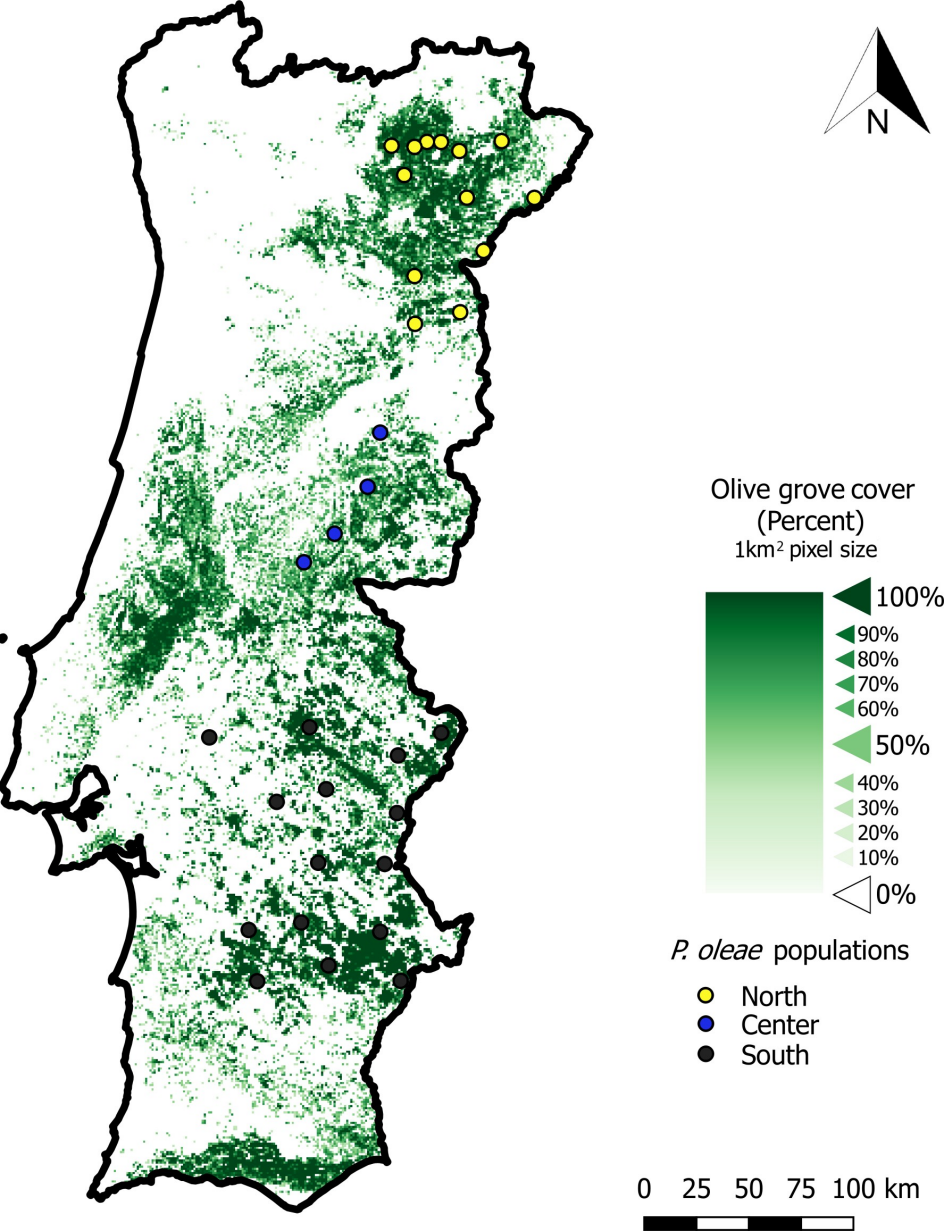
*Prays* sampling sites (colored dots), discriminated by the three regions (North, Center and South). Green color on background represents the density of olive groves per square kilometer, ranging from low density (light green) to high density (dark green).

No specific permissions are required to sample olive moth. All the samples were obtained from the monitoring services of the Portuguese Ministry of Agriculture or from private land with the permission of the owners and did not involve endangered or protected species. Biological material was collected from 28 sites (Fig 1) using commercial sticky traps with specific pheromones (Biosani) during the summer of 2017. The installation and collection of the traps was partially performed by local associations and/or by the Regional Services for Agriculture (S1 Table). The traps stayed for at least a week at the designated olive locations, most of them used to monitoring the pest, and were then transported to the laboratory in individual plastic bags. The trapped adults, putatively belonging to *Prays oleae*, were collected from the traps and stored at -20°C in 70% ethanol until DNA extraction. Individuals were allowed to dry on filter paper prior to DNA extraction. DNA from whole body tissue was extracted following extraction protocols using CTAB extraction buffer [14] after being ground up with a plastic pestle. Proteins were removed with 24 : 1 isoamylalcohol : chloroform, and DNA precipitated with isopropanol. DNA extracts were eluted in 50 μL of sterile water. All extraction products were stored at -20°C and later used directly in the PCR.

### *Prays* sequences

Two mitochondrial genes (*COI* and *nad5*) and one protein-coding nuclear gene region (*RpS5*) were amplified using the following primer pairs: 1) LCO1490 (5’- GGT CAA CAA ATC ATA AAG ATA TTG G -3' and HCO2198 (5’- TAA ACT TCA GGG TGA CCA AAA AAT CA -3’) for a fragment of the cytochrome c oxidase subunit I (*COI*) gene [15]; 2) nad5_fw (5’- TTA TAT CCT TAG AAT AAA ATC C -3’) and nad5_rev (5’- TTA GGT TGA GAT GGT TTA GG -3’) for a fragment of the NADH dehydrogenase subunit 5 (*nad5*) gene [16] and 3) RpS5_f (5’- ATG GCN GAR GAR AAY TGG AAY GA -3’) and RpS5_r (5’- CGG TTR GAY TTR GCA ACA CG -3’) for a fragment of the ribosomal protein S5 (*RpS5*) gene [17]. PCR reactions were conducted using 1 μl of the extracted DNA in a standard 25 μl reaction, with 0.5 pmol/μl of each primer, 1.5 mM MgCl^2^, 0.5 mM dNTPs and 0.04 U/ml Taq DNA polymerase. The cycle protocol involved initial denaturation at 94°C for 2 min, followed by 30 cycles of 94°C for 30 s, gene-specific annealing temperatures (55°C for COI and nad5, and 53°C for RpS5) for 30 s and 72°C for 1 min, and an extension cycle of 72°C for 7 min; the PCR product was purified using the NZYGelpure kit (from NZYTech, Lda) and sequencing was done commercially (Macrogen Inc.). The sequences were assembled, edited and aligned using the CLC Main Workbench version 7.5.1 (Qiagen Aarhus A/S, Denmark) (all sequences generated in this study and their GenBank accession numbers are in S2 Table). The COI sequences obtained in this study were organized as haplotypes, and a subset of representative COI haplotypes were selected based on high level of differentiation (no. of differences) and on their representativeness in the sample. These were combined with those available for the genus *Prays* from GenBank to generate a global dataset.

### Phylogenetic analysis

The phylogenetic reconstruction analysis based on the COI sequences was performed in BEAST v.4.2.8 [18]. We selected the Gamma Site Model with 4 gamma categories, and rate frequencies were estimated. All other settings were left as default, including the chain length of 10 000 000 generations. The output of BEAST was analysed in the software Tracer v.1.6 to determine chain convergence and burnin. The majority rule consensus tree was obtained from the trees sampled in the analysis using the program TreeAnnotator v.2.4.8, considering a burn-in of 10% (first 1000 trees were removed). Reconstructions with Maximum Likelihood and Neighbor-Joining methods as implemented in Mega 7.0 [19] were performed for testing for congruence between methods (S1 and S2 Figs). To test the monophyly of *Prays oleae*, we inferred the phylogeny once without constraints and once with all accessions of *P*. *oleae* constrained to be monophyletic, to evaluate the likelihood of this alternative phylogenetic relationship. Bayes factors were used to test if the topological constrained topology was significantly different than the unconstrained topology, and was measured using twice the difference of −ln likelihood (2lnBF) with 2lnBF = 0–2 meaning not worth a mention, 2lnBF = 2–6 meaning positive support, 2lnBF = 6–10 meaning strong support, and 2lnBF > 10 meaning decisive support [20].

### Variability and population structure

Sequence variability analyses of the three DNA fragments analysed were performed in DnaSP v. 4.0 [21]. For haplotype and nucleotide diversity estimates (Hd and Pi), we chose to analyze synonymous and non-synonymous sites jointly because if analyzed separately, the number of sites would have been too low to yield reliable results [22]. Tajima’s D statistics compares the average number of pairwise differences with the number of segregating sites [23]. Over the all sequenced fragments, linkage disequilibrium was measured using the ZnS statistic (the squared allele frequency correlation r^2^ [24]) on the basis of the parsimony informative sites. Statistical significance for ZnS and Tajima’s D was assessed by coalescent simulations with 10 000 replicates as implemented in DnaSP v. 4.0 [21], conducted considering all segregating sites and an intermediate level of recombination. These analyses were performed for the sequence data of the three amplicons independently and concatenated. A haplotype network approach was chosen for a concise representation of the dataset obtained in this work, both concatenated and all three regions separately. The haplotype networks were constructed in PopART [25] using TCS network (95% connection limit).

### Ecological niche modelling

Georeferenced *Prays oleae* capture sites were used to obtain Ecological Niche Models (ENM’s) employing the maximum entropy algorithm in Maxent 3.4.0 (Maximum entropy modeling of species geographic distributions). Predictions were based on a set of 16 environmental data maps including bioclimatic [26] and land cover derived maps (S3 Table). This information was prepared for the area where *Prays* captures were registered (NUTS3 European administrative limits were considered) and exported to ASCII grid format with 1 km^2^ resolution using QGIS [27]. Among highly correlated covariables (Pearson correlation coefficient R > 0.75, ENMTools 1.4.4 [28,29]) only the ones presenting the highest percentage of importance to the model in a preliminary run was retained for further procedures [30] (S3 Table). Prediction models were run for 10 interactions using 50% random records to test each run, and the model quality was accessed by area under the curve (AUC scores above 0,7 are acceptable for a good model performance, [31]).

For the main *Prays* groups (defined in the phylogenetic analyses), the ENM’s similarity was accessed by calculating Schoener’s D [32] and Hellinger’s I [29] indices and then preforming an identity test (run with 300 pseudoreplicates, ENMTools 1.4.4 [28]).

## Results

**Fig 2:**
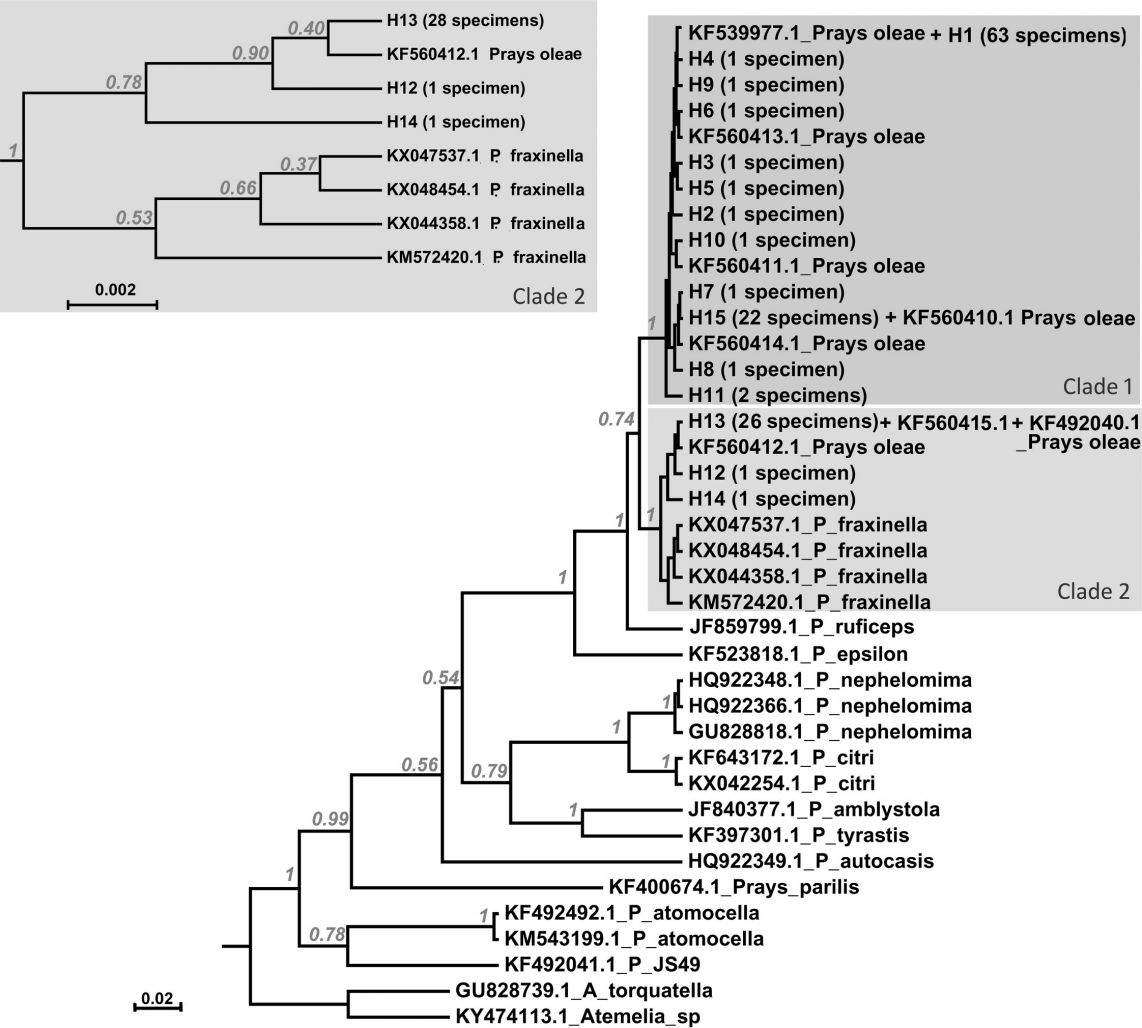
Phylogenetic relationship between *Prays oleae* and other *Prays* species with data available on GenBank (accession code given on tree), based on the COI amplicon. The phylogeny corresponds to the majority rule consensus tree of trees sampled in a Bayesian analysis, and the posterior probability values are shown for main nodes (xml input files available in S1 File). Two *Atemelia* species were used as outgroups. Similar topologies were obtained using Maximum Likelihood and Neighbor-Joining methods as implemented in Mega 7.0 [19] (S1 and S2 Figs) and will further not be discussed. The top left grey window highlights a clade showing *Prays oleae* specimens nesting in the *Prays fraxinella* clade, an unexpected result. *Prays oleae* samples were collected from Portugal with exception of KF560413.1, KF560414.1, and KF560415.1 which were collected in Tunisia and KF492040.1 from Spain. The tip labels represent either the haplotypes and number of specimens with that same haplotype (details in S2 Table) or the GenBank accession number.

The reconstructed unconstrained phylogeny of the genus *Prays* based on COI (Fig 2) resolved *Prays oleae* as paraphyletic. This model is significantly better than when monophyly of *P*. *oleae* samples is constrained (2·*ln*BF = 33.17; with BI *ln* [unconstrained model] = -2830.90 and BI *ln* [alternative model] = -2847.49).

The three amplicons–*COI*, *nad5* and *RpS5*– were sequenced for 128 specimens spanning relevant olive groves areas in Portugal and their variability (number of haplotypes, polymorphic sites and diversity estimates) is presented in Table 1. Because of the observed non-monophyly of *Prays oleae* (Fig 2), we present the same estimates separately for the samples nested in clade 1 or in clade 2 (Table 1). Levels of nucleotide diversity for the mtDNA amplicons are equivalent between sequenced regions, with the numbers of non-synonymous changes being half of the synonymous ones. An exception to this is in clade 2, where the two mitochondrial regions behave differently (Table 1). Contributing to this might be the low number of samples analysed. The fragment of the nuclear gene encoding for the ribosomal protein S5a (RpS5) shows almost equivalent numbers of non-synonymous and synonymous sites (except for clade 2; again low number of samples needs to be kept in mind). Considering the full dataset together, no linkage disequilibrium was detected and the Tajima’s D statistics was non-significant for all markers suggesting that these DNA sequences have evolved randomly (‘neutrality’) (Table 2). When analyzing clade 1 and clade 2 separately, the Tajima’s D statistics for the mitochondrial COI marker is in both cases significant (Table 2).

**Table 1:** Sequence variability analyses of the three DNA fragments analysed, considering the complete dataset and partitioned by clade as identified in Fig 2.

	COI	nad5	RpS5	all markers
**Number of sequences**	128	128	128	128
**Number of sites (bp)**	620	676	517	1813
**Number of haplotypes**	26	31	19	85
**Polymorphic sites (S)**	43	41	22	106
Parsimony informative	21	25	8	54
**Total number of mutations**	46	41	22	109
Synonymous changes	32	27	12	71
Non-Synonymous	14	14	10	38
**Haplotype diversity (Hd)**	0.754	0.891	0.733	0.984
**Aver. nucleotide diff. (k)**	6.243	7.435	1.857	15.536
**Nucleotide diversity (Pi)**	0.010	0.011	0.004	0.008
*Prays oleae* Clade 1				
**Number of sequences**	101	101	101	101
**Number of haplotypes**	23	27	17	68
**Polymorphic sites (S)**	23	39	16	78
Parsimony informative	7	21	7	35
**Total number of mutations**	23	39	16	78
Synonymous changes	16	27	9	52
Non-Synonymous	7	12	7	26
**Haplotype diversity (Hd)**	0.663	0.870	0.752	0.983
**Aver. nucleotide diff. (k)**	1.698	4.176	1.827	7.701
**Nucleotide diversity (Pi)**	0.003	0.006	0.003	0.004
*Prays oleae* Clade 2				
**Number of sequences**	27	27	27	27
**Number of haplotypes**	3	7	9	17
**Polymorphic sites (S)**	12	23	12	47
Parsimony informative	0	20	3	23
**Total number of mutations**	12	22	12	46
Synonymous changes	5	20	8	33
Non-Synonymous	7	2	4	13
**Haplotype diversity (Hd)**	0.145	0.601	0.561	0.869
**Aver. nucleotide diff. (k)**	0.889	5.322	1.692	7.903
**Nucleotide diversity (Pi)**	0.002	0.008	0.003	0.004

**Table 2:** Population genetics inferences based on Tajima's D, the site frequency spectrum (SFS) of mutations; and ZnS, the statistical association among those (linkage disequilibrium). The same statistics are presented for the dataset portioned by clade as identified in Fig 2.

	Tajima´s D	Significance*	ZnS	Significance*
All *Prays oleae* sequences (n = 128)				
**COI**	-0.650	p = 0.26; [-1.54, 1.94]	0.123	p = 0.61; [-0.03, 0.34]
**nad5**	-0.049	p = 0.56; [-1.53, 1.88]	0.118	p = 0.59; [0.04, 0.31]
**RpS5**	-1.544	p = 0.01; [-1.32, 1.57]	0.076	p = 0.592; [0.03, 0.16]
**All markers**	-0.660	p = 0.19; [-1.19, 1.14]	0.062	p = 0.297; [0.04, 0.14]
*Prays oleae* Clade 1 (n = 101)				
**COI**	-1.818	p = 0.00*; [-1.36, 1.44]	0.018	p = 0.00*; [-0.03, 0.16]
**nad5**	-1.385	p = 0.02; [-1.34, 1.42]	0.095	p = 0.74; [0.04, 0.15]
**RpS5**	-1.139	p = 0.07; [-1.37, 1.66]	0.063	p = 0.38; [0.03, 0.20]
**All markers**	-1.589	p = 0.00*; [-1.37, 1.66]	0.033	p = 0.00*; [0.04, 0.15]
*Prays oleae* Clade 2 (n = 27)				
**COI**	-2.386	p = 0.00*; [-1.45, 1.49]	0.834	p = 1.00; [0.06, 0.34]
**nad5**	-0.389	p = 0.31; [-1.41, 1.37]	0.631	p = 1.00; [0.07, 0.27]
**RpS5**	-1.524	p = 0.02; [-1.43, 1.50]	0.212	p = 0.85; [0.06, 0.33]
**All markers**	-1.332	p = 0.01; [-1.27, 1.35]	0.222	p = 0.94; [0.08, 0.26]

*p-value; 99% confidence interval

**Fig 3:**
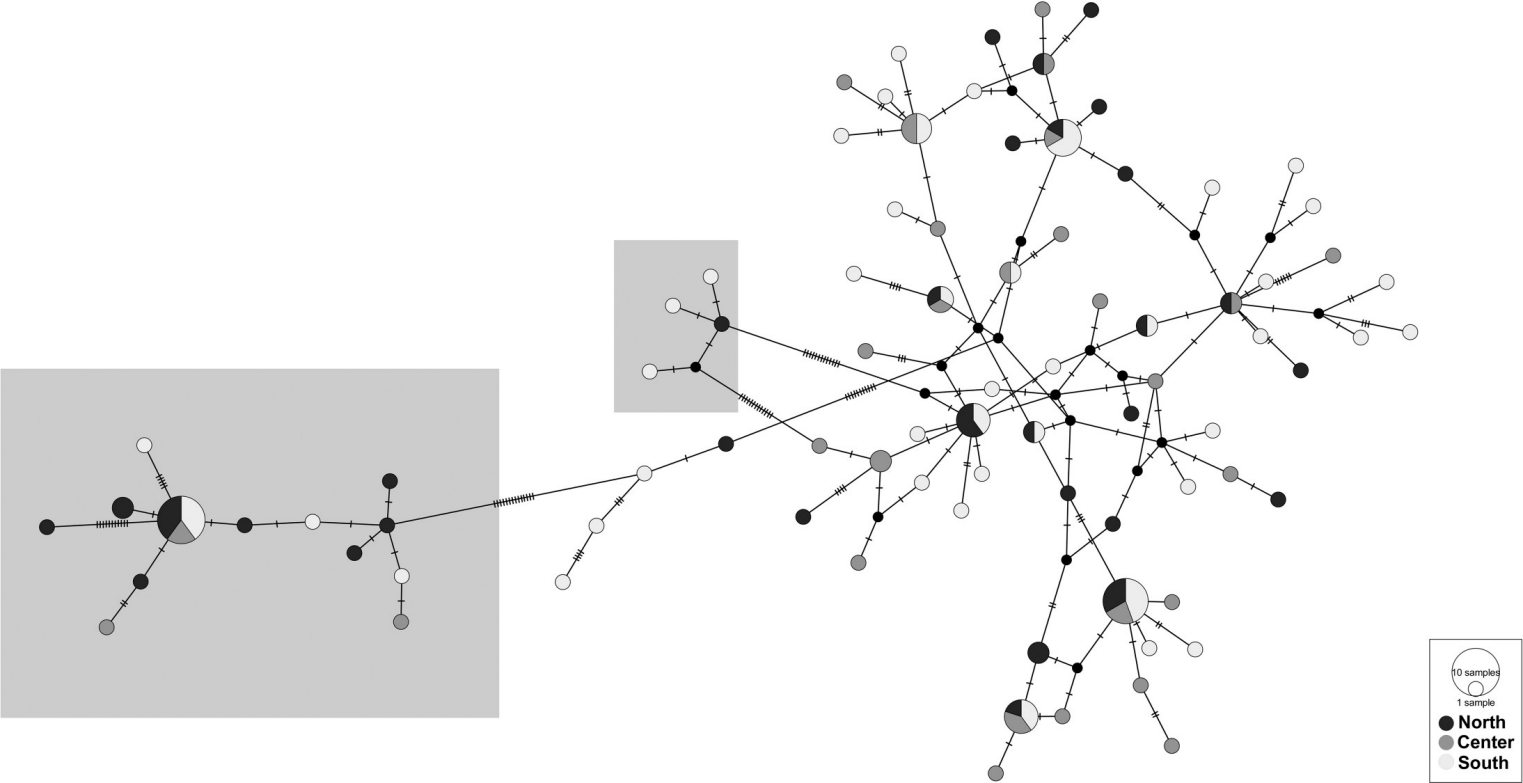
TCS haplotype networks based on the three markers (COI, nad5 and RpS5). Each circle represents a sequence; the size of the circle is proportional to number of individuals with a particular haplotype sequence. The connections are mutational steps between individuals. The grey windows highlight the *Prays oleae* clade 2 (Fig 2).

The haplotype network showed a complex and diversified topology, consisting of one main star-like arrangement with satellites and a slightly more complex adjacent network (Fig 3, haplotype networks partitioned per gene are presented in S3 Fig). No obvious relation with sampling location (North, Center or South) is observed, being the clearest pattern coming from the two *P*. *oleae* clades previously identified in Fig 2.

Maxent ENM’s for both clades showed a good predictive ability (clade 1, test AUC ± SD = 0.80 ± 0.07; clade 2, test AUC ± SD = 0.86 ± 0.07). The percentage contribution of each predictor showed, as obviously expected, that “distance to homogeneous olive groves (> 1 ha)” had the greatest contribution to clade 1 (79.5%) and clade 2 (87.7%) models, being followed by continentally (in one or another index form; Table 3). The overlap indices obtained for clade 1 and clade 2 models (Schoener’s D = 0.72, Hellinger’s I = 0.94) were not inferior to the null distribution of 300 pseudoreplicates (Schoener’s D $\bar{x}$ ± SD = 0.71 ± 0,04,Hellinger’s I $\bar{x}$ ± SD = 0.92 ± 0,02), indicating identical niche models.

**Table 3:** Relative importance to ENM-variables for both identified *Prays oleae* clades. The absence of contribution score (-) means the variable was not included in the respective clade final model.

Variables description	Contribution (%)	
	clade 1	clade 2
Distance to homogeneous olive groves (> 1 ha)	79.5	87.7
Simple continentality (Rivas-Martínez 2007, 2008, 2011)	11.6	-
Distance to riparian gallery	5.5	-
Mean temperature of the warmest month of the year	1.8	-
Density of olive groves per km^2^	1.5	3.2
Simple continentality index	-	4.8

## Discussion

The unconstrained topology (Fig 2) resolves *Prays oleae* as non-monophyletic, questioning its’ species status. Constraining the olive moth to monophyly resulted in a topology significantly worse than the unconstrained one. From the samples obtained in the present work, about one third forms a well-supported clade with *Prays fraxinella* (clade 2), while the rest is sister to that clade (clade 1) (Fig 2). Only eight records of *Prays oleae* were available for the cytochrome c oxidase subunit I (COI) gene, comprising samples from Portugal (4), Tunisia (3) and Spain (1), but specimens belonging to both *Prays oleae* putative clades were found in all three countries suggesting the existence of two sympatric evolutionary lineages of cryptic olive moths with no described phenotypical differences.

However, we need to acknowledge the possibility that the distinct mitochondrial subdivision observed in the partial COI gene fragment of *P*. *oleae* might not be well corroborated by other DNA sequences (particularly nuclear), genital morphology, mating behaviour or ecological niche. Actually, and contrary to our hypothesis, the observed non-monophyly of *Prays oleae* does not seems to be reflected in a different use of the habitat by the specimens belonging to the identified clades (the niche modelling showed no niche differentiation for the variables used). If we are indeed dealing with two differentiated lineages, they either have the same ecological niche or we were unable to identify the divergent axis of their niche.

Also population statistics were congruent regardless of grouping the samples by *P*. *oleae* clade. Tajima’s D statistics provided the main exception, with the mitochondrial marker COI being significant only when analyzing the two clades separately. The significant negative value of Tajima’s D suggests either a recent selective sweep (or linkage to it) or a recent population expansion following a bottleneck, as these values are negative when there is an excess of rare variants. The fact that this was only observed for the COI marker and that the impact of difference in sample size on estimates of Tajima’s D is difficult to track analytically (because both sampling strategy and species demographic history can have impact on the estimate; see, for example [33]), we cannot conclude on population dynamics.

The selection of markers for a given analyses is critical for results interpretation. For phylogenetic studies, the advantages of mitochondrial DNA are well known: 1) strict maternal transmission; 2) high mutation rate and 3) conserved simple structure, allowing the design of “universal” primers. Protein coding genes seem to be the most useful when dealing with taxonomic levels such as families, genera and species, and amongst these, the *COI* is found to be the best and most widely used molecular marker for DNA barcoding, species identification and evolutionary studies [34]. In the case of the present study, and based on the reported reliability of the *COI* gene, we could conclude that *P*. *oleae* variability and its non-monophyly suggest cryptic species diversity questioning the phylogenetic relation between *P*. *oleae* and other *Prays* species, in particular with *P*. *fraxinella*. However, and considering the results and constraints presented, any conclusions require caution. A taxonomic revision is doubtlessly needed, as a correct identification is the basis for the management of species in the ecosystem and hence crucial for risk assessment and pest control in the agricultural context. Taxon sampling influences phylogenetic inferences, and it should be broadened to include more representatives of the species within the genus *Prays* and from a wider geographical area. While no complete genomes and only the *Prays oleae* mitogenome are available [35], the phylogeny within this genus needs to be tackled through the analyses of more molecular markers than the *COI* gene, towards an understanding of speciation and eventual hybridizations.

Molecular genetic methods have been uncovering cryptic lineages [22,36–38] but the extent to which this genetic variation affects phenotypic traits is unclear. The genetic divergence observed within cryptic lineage complexes may result in differences other than morphological ones: traits related to intraspecific competition and predator avoidance, for instance, can vary, making generalizations regarding their response to environmental variables inappropriate if made assuming one single lineage [39]. In the case of the olive moth, a recognized pest of olives, the foretold existence of two cryptic lineages with potential deviation in traits might have a high impact in the agro-ecosystem management. It urges thus to confirm the findings of the present work, by expanding the sampling throughout the species distribution. Furthermore, the disclosure of lineage-specific differences in biological traits between the identified lineages is fundamental for the development of appropriate pest management practices.

## Supporting information

S1 Fig Phylogenetic relationship between *Prays oleae* and other *Prays* species with data available on GenBank (accession code given on tree), based on the COI amplicon, by Maximum Likelihood method. (PDF)

S2 Fig Evolutionary relationships between *Prays oleae* and other *Prays* species with data available on GenBank (accession code given on tree), based on the COI amplicon, by Neighbor-Joining. (PDF)

S3 Fig TCS haplotype networks of sampled specimens, considering a) the mitochondrial marker COI, b) the mitochondrial marker nad5, and c) the nuclear marker RpS5. Each circle represents a sequence; the size of the circle is proportional to number of individuals with a particular haplotype sequence.
(PDF)

S1 Table Sampled locations and geographical coordinates. Locations with alphabetic code only were sampled by local associations and/or by the Regional Directorates for Agriculture.
(PDF)

S2 Table GenBank accession numbers of the sequences obtained in this study. Code corresponds to the location code as in Table 1 followed by specimen number.
(PDF)

S3 Table Variables analysed, including bioclimatic and land cover derived maps. (PDF)

S1 File Compressed folder containing 4 data files: Fasta file with *Prays* sequences alignment, Newick files of S1 and S2 Figs and the BEAST xml constrained and unconstrained files. (ZIP)
