# Supplementary material for: Uncovered variability in olive moth (Prays oleae) questions species monophyly
Source: PLoS One. 2018 Nov 26;13(11):e0207716. doi: 10.1371/journal.pone.0207716 (PMC6261264; doi:10.1371/journal.pone.0207716)
Supplement: S1 Fig — (PDF) [file pone.0207716.s001.pdf]

Supplemental information

Uncovered variability in olive moth (*Prays oleae*) questions species monophyly

Tânia Nobre[(1)]*, Luis Gomes[(1)], Fernando Trindade Rei[(1)]
[(1)] Laboratory of Entomology, ICAAM, University of Évora
* Corresponding author: tnobre@uevora.pt

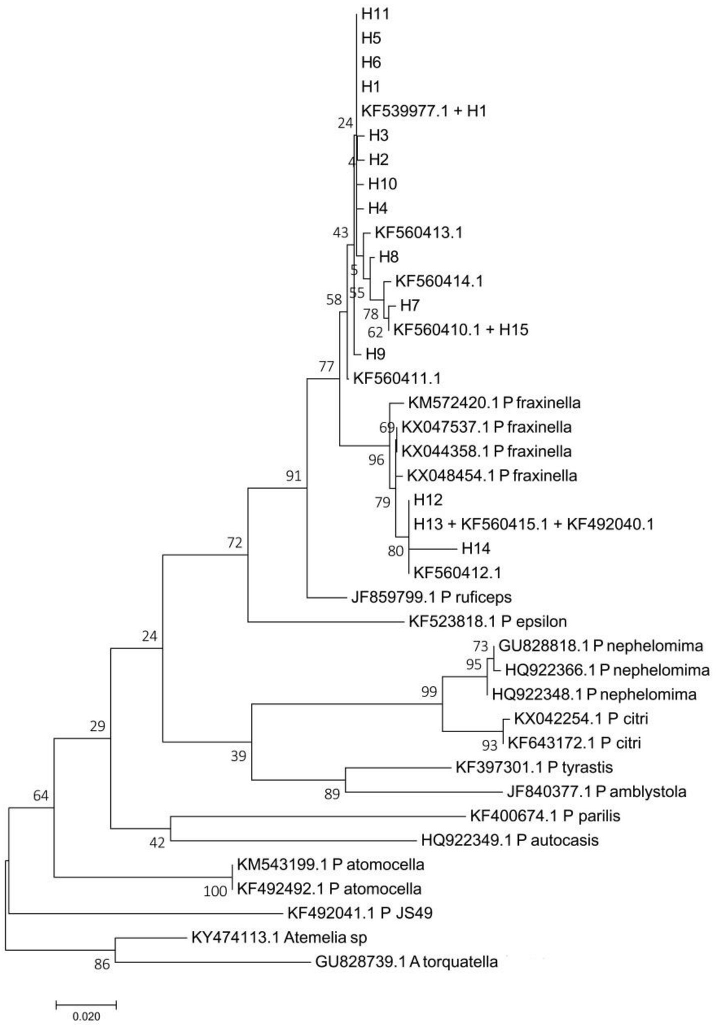

S1 Fig. Phylogenetic relationship between *Prays oleae* and other *Prays* species with data available on GenBank (accession code given on tree), based on the *COI* amplicon, by Maximum Likelihood method**.** See *Prays_alignment_COI.fa* and *COIPrays_MLtree.nwk* in S1 File.

The evolutionary history was inferred by using the Maximum Likelihood method based on the General Time Reversible model. The percentage of trees in which the associated taxa clustered together is shown next to the branches. Initial tree(s) for the heuristic search were obtained automatically by applying Neighbor-Join and BioNJ algorithms to a matrix of pairwise distances estimated using the Maximum Composite Likelihood (MCL) approach, and then selecting the topology with superior log likelihood value. A discrete Gamma distribution was used to model evolutionary rate differences among sites (4 categories (+*G*, parameter = 0.3085)). The tree is drawn to scale, with branch lengths measured in the number of substitutions per site. See main text for details.
