# Supplementary material for: Uncovered variability in olive moth (Prays oleae) questions species monophyly
Source: PLoS One. 2018 Nov 26;13(11):e0207716. doi: 10.1371/journal.pone.0207716 (PMC6261264; doi:10.1371/journal.pone.0207716)
Supplement: S2 Fig — (PDF) [file pone.0207716.s002.pdf]

Supplemental information

Uncovered variability in olive moth (*Prays oleae*)
questions species monophyly

Tânia Nobre[(1)]*, Luis Gomes[(1)], Fernando Trindade Rei[(1)]
[(1)] Laboratory of Entomology, ICAAM, University of Évora
* Corresponding author: tnobre@uevora.pt

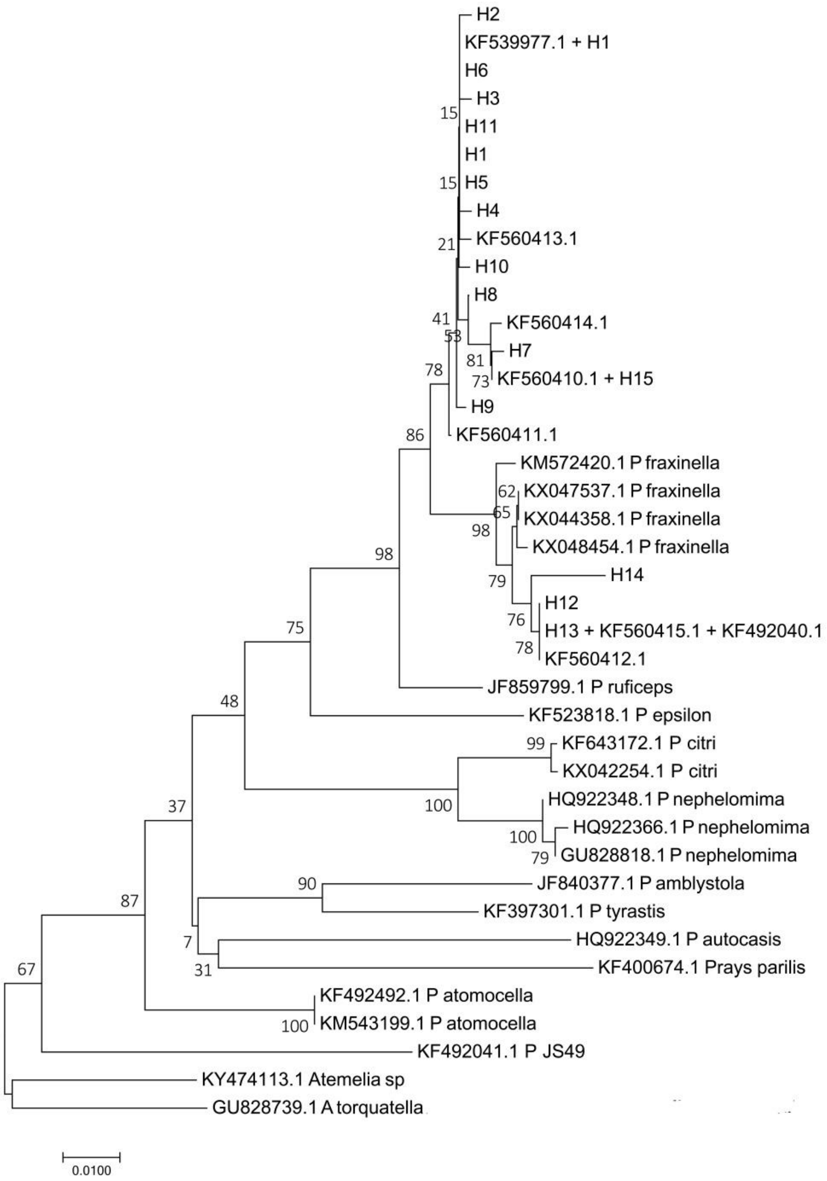

S2 Fig. Evolutionary relationships between *Prays oleae* and other *Prays* species with data available on GenBank (accession code given on tree), based on the *COI* amplicon, by Neighbor-Joining. See *Prays_alignment_COI.fa* and *COIPrays_NJtree.nwk* in S1 File.

The evolutionary history was inferred using the Neighbor-Joining method. The percentage of replicate trees in which the associated taxa clustered together in the bootstrap test (1000 replicates) are shown next to the branches. The tree is drawn to scale, with branch lengths in the same units as those of the evolutionary distances used to infer the phylogenetic tree. The evolutionary distances were computed using the Maximum Composite Likelihood method and are in the units of the number of base substitutions per site. See main text for details.
