# Supplementary material for: Uncovered variability in olive moth (Prays oleae) questions species monophyly
Source: PLoS One. 2018 Nov 26;13(11):e0207716. doi: 10.1371/journal.pone.0207716 (PMC6261264; doi:10.1371/journal.pone.0207716)
Supplement: S3 Fig — TCS haplotype networks of sampled specimens, considering a) the mitochondrial marker COI, b) the mitochondrial marker nad5, and c) the nuclear marker RpS5. Each circle represents a sequence; the size of the circle is proportional to number of individuals with a particular haplotype sequence. (PDF) [file pone.0207716.s003.pdf]

Supplemental information

Uncovered variability in olive moth (*Prays oleae*) questions species monophyly

Tânia Nobre[(1)]*, Luis Gomes[(1)], Fernando Trindade Rei[(1)]
[(1)] Laboratory of Entomology, ICAAM, University of Évora
* Corresponding author: tnobre@uevora.pt

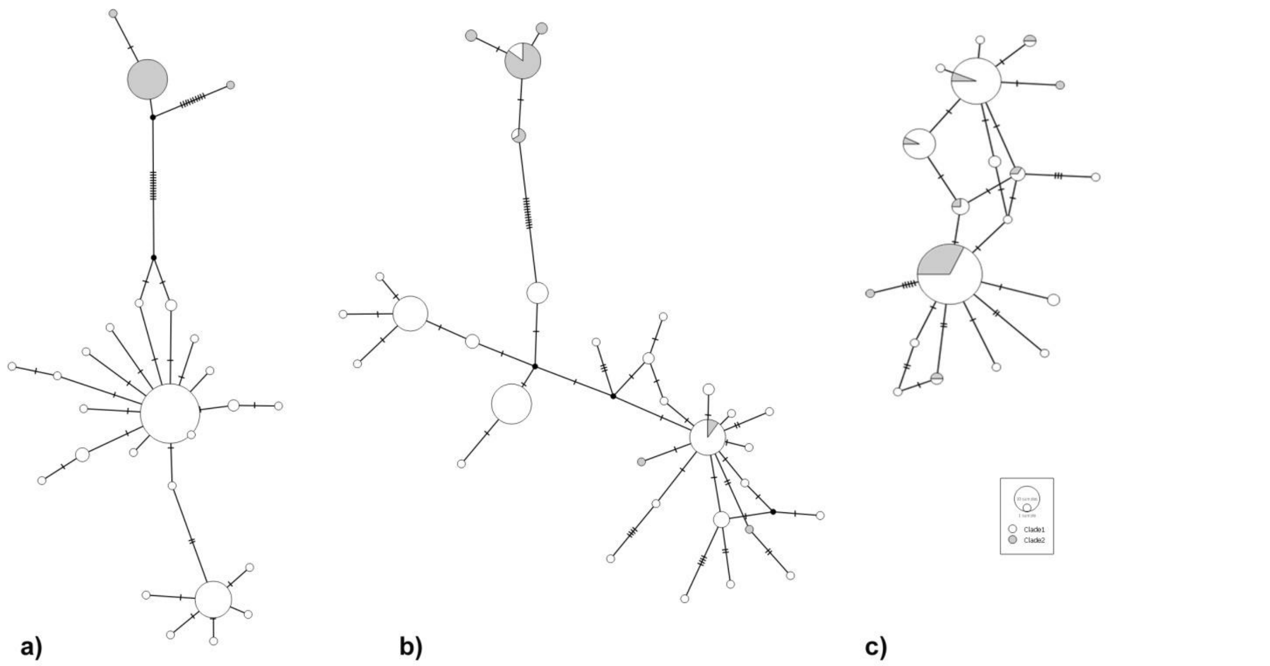

S3 Fig: TCS haplotype networks of sampled specimens, considering a) the mitochondrial marker COI, b) the mitochondrial marker nad5, and c) the nuclear marker RpS5. Each circle represents a sequence; the size of the circle is proportional to number of individuals with a particular haplotype sequence. The connections are mutational steps between individuals. The grey colour shows the proportion of individuals on clade 2 based on the COI amplicon (Fig 2).
