# Supplementary material for: Uncovered variability in olive moth (Prays oleae) questions species monophyly
Source: PLoS One. 2018 Nov 26;13(11):e0207716. doi: 10.1371/journal.pone.0207716 (PMC6261264; doi:10.1371/journal.pone.0207716)
Supplement: S1 Table — Locations with alphabetic code only were sampled by local associations and/or by the Regional Directorates for Agriculture. (PDF) [file pone.0207716.s004.pdf]

<u>Supplemental information</u>

Uncovered variability in olive moth (*Prays oleae*) questions species monophyly

Tânia Nobre[(1)]*, Luis Gomes[(1)], Fernando Trindade Rei[(1)]
[(1)] Laboratory of Entomology, ICAAM, University of Évora
* Corresponding author: tnobre@uevora.pt

S1 Table: Sampled locations and geographical coordinates. Locations with alphabetic code only were sampled by local associations and/or by the Regional Directorates for Agriculture.

Closest locality	Coord X	Coord y	Code	Region
Baleizão	-7.708961	38.013240	**P15**	South
Telheiro	-7.398881	38.448920	**P86**	South
Ervidel	-8.097120	37.945810	**P4**	South
Mina do Bugalho	-7.328936	38.667503	**P139**	South
Moura	-7.425753	38.156567	**P52**	South
Vila de Frades	-7.856793	38.198422	**P61**	South
Odivelas	-8.143498	38.166765	**P71**	South
Vila Verde de Ficalho	-7.316707	37.944818	**P24**	South
Vila Fernando	-7.320529	38.915488	**P170**	South
Évora Monte	-7.717580	38.772255	**P129**	South
Campo Maior	-7.080551	39.011178	**P180**	South
Ervedal	-7.807832	39.039536	**P162**	South
Cabeção	-8.069177	38.948084	**P149**	South
Couço	-8.361094	38.995884	**P155**	South
São Manços	-7.762467	38.455650	**P79b**	South
Arraiolos	-7.989730	38.718441	**P119**	South
Sarzedas	-7.663531	39.872450	**SAR**	Center
Sobreira Formosa	-7.835537	39.749912	**GSF**	Center
Castelo Novo	-7.477043	40.073788	**CNV**	Center
Lamaçais	-7.404250	40.306381	**LMC**	Center
Pinhel	-7.111541	40.727906	**PNH**	North
Meda	-7.275059	40.951070	**MDA**	North
Vilar Torpim	-7.002735	40.901195	**VTM**	North
Mogadouro	-6.512559	41.308013	**MGD**	North
S. Salvador	-7.193772	41.535096	**SSR**	North
F.E.Cinta	-7.431884	41.049872	**FEC**	North
Torre Moncorvo	-7.041674	41.554825	**TMC**	North
Sendim da Ribeira	-6.899215	41.327122	**SRB**	North
F. Mercê	-7.325467	41.540939	**FMC**	North
Izeda	-6.694296	41.561408	**IZD**	North
Cobro	-7.246815	41.414660	**CBR**	North
Paradela	-7.120856	41.554680	**PDL**	North
M. Caval / Castelões	-6.937525	41.514888	**MCL**	North
