# Supplementary material for: Uncovered variability in olive moth (Prays oleae) questions species monophyly
Source: PLoS One. 2018 Nov 26;13(11):e0207716. doi: 10.1371/journal.pone.0207716 (PMC6261264; doi:10.1371/journal.pone.0207716)
Supplement: S2 Table — Code corresponds to the location code as in Table 1 followed by specimen number. (PDF) [file pone.0207716.s005.pdf]

Supplemental information

Uncovered variability in olive moth (*Prays oleae*) questions species monophyly

Tânia Nobre[(1)]*, Luis Gomes[(1)], Fernando Trindade Rei[(1)]
[(1)] Laboratory of Entomology, ICAAM, University of Évora
* Corresponding author: tnobre@uevora.pt

S2 Table: GenBank accession numbers of the sequences obtained in this study. Code corresponds to the location code as in Table 1 followed by specimen number.

Code	*	COI	nad5	RpS5
CBR1	*H1*	MH536341	MH536482	MH550668
CBR2	*H1*	MH536342	MH536479	MH550735
CBR3	*H1*	MH536343	MH536482	MH550736
CBR5	*H1*	MH536344	MH536505	MH550737
CBR6	*H13*	MH536446	MH536482	MH550669
CNV4	*H17*	MH536411	MH536477	MH550670
CNV5	*H1*	MH536345	MH536477	MH550671
CNV6	*H1*	MH536346	MH536486	MH550672
FEC2	*H15*	MH536420	MH536482	MH550738
FEC3	*H1*	MH536347	MH536482	MH550673
FEC4	*H1*	MH536348	MH536473	MH550739
FEC5	*H1*	MH536349	MH536473	MH550674
FEC6	*H16*	MH536350	MH536493	MH550740
FMC2	*H1*	MH536353	MH536477	MH550741
FMC3	*H13*	MH536447	MH536502	MH550675
FMC4	*H9*	MH536412	MH536495	MH550742
GSF1	*H1*	MH536354	MH536477	MH550743
GSF2	*H1*	MH536355	MH536482	MH550744
GSF3	*H1*	MH536356	MH536487	MH550676
GSF4	*H1*	MH536357	MH536476	MH550677
GSF6	*H13*	MH536448	MH536504	MH550745
IZD1	*H13*	MH536449	MH536502	MH550678
IZD2	*H15*	MH536421	MH536473	MH550746
IZD3	*H10*	MH536410	MH536477	MH550747
IZD5	*H1*	MH536358	MH536476	MH550748
IZD6	*H1*	MH536359	MH536482	MH550749
LMC1	*H1*	MH536360	MH536501	MH550679
LMC2	*H18*	MH536361	MH536477	MH550750
LMC3	*H2*	MH536415		
LMC4	*H1*	MH536362	MH536482	MH550751
LMC5	*H1*	MH536363	MH536495	MH550752
LMC6	*H1*	MH536364	MH536486	MH550680
MCL1	*H13*	MH536450	MH536502	MH550753
MCL2	*H13*	MH536451	MH536502	MH550754
MCL3	*H15*	MH536422	MH536473	MH550755

Code	*	COI	nad5	RpS5
MDA3	*H19*	MH536365	MH536500	MH550756
MDA4	*H1*	MH536367	MH536474	MH550757
MDA5	*H4*	MH536368	MH536486	MH550681
MDA6	*H1*	MH536369	MH536477	MH550758
MGD1	*H15*	MH536423	MH536473	MH550682
MGD2	*H15*	MH536424	MH536473	MH550683
MGD6	*H1*	MH536370	MH536498	MH550684
P119A	*H6*	MH536371	MH536477	MH550759
P119B	*H1*	MH536372	MH536482	MH550685
P119D	*H1*	MH536413	MH536484	MH550791
P119E	*H1*	MH536373	MH536474	MH550686
P129A	*H1*	MH536442	MH536473	MH550687
P129C	*H15*	MH536425	MH536473	MH550688
P129F	*H15*	MH536426	MH536473	MH550689
P139A	*H1*	MH536374	MH536477	MH550760
P139C	*H16*	MH536351	MH536486	MH550761
P139D	*H13*	MH536452	MH536492	MH550690
P139E	*H20*	MH536443	MH536502	MH550691
P139F	*H1*	MH536375	MH536473	MH550692
P155A	*H1*	MH536376	MH536502	MH550762
P15A	*H1*	MH536377	MH536482	MH550763
P15B	*H13*	MH536453	MH536502	MH550732
P15E	*H13*	MH536454	MH536502	MH550693
P15F	*H1*	MH536378	MH536477	MH550764
P162B	*H15*	MH536427	MH536473	MH550765
P162D	*H1*	MH536428	MH536473	MH550766
P170D	*H1*	MH536379	MH536482	MH550694
P170E	*H1*	MH536380	MH536496	MH550767
P170F	*H13*	MH536455	MH536502	MH550695
P180A	*H1*	MH536381	MH536491	MH550768
P180B	*H3*	MH536382	MH536481	
P180C	*H1*	MH536383	MH536477	MH550696
P180D	*H21*	MH536409	MH536482	MH550769
P180F	*H1*	MH536384	MH536477	MH550697
P24B	*H13*	MH536456	MH536502	MH550698
P24C	*H22*	MH536385	MH536484	MH550770
P24D	*H1*	MH536386	MH536485	MH550699
P24E	*H23*	MH536444	MH536475	MH550700
P24F	*H1*	MH536387	MH536482	MH550787
P4A	*H1*	MH536388	MH536474	MH550771
P4C	*H13*	MH536457	MH536504	MH550772
P4D	*H1*	MH536389	MH536483	MH550773
P52D	*H13*	MH536458	MH536488	MH550701
P52E	*H1*	MH536414	MH536477	MH550702
P52F	*H12*	MH536459	MH536482	MH550703
P61A	*H15*	MH536429	MH536502	MH550774

Code	*	COI	nad5	RpS5
P61B	*H1*	MH536390	MH536477	MH550775
P61D	*H1*	MH536391	MH536502	
P61E	*H11*	MH536392	MH536477	MH550704
P61F	*H1*	MH536417	MH536482	MH550705
P71A	*H24*	MH536393	MH536482	MH550706
P71C	*H19*	MH536366	MH536477	MH550707
P71E	*H1*	MH536394	MH536482	MH550708
P79bA	*H15*	MH536430	MH536473	MH550709
P79bB	*H1*	MH536395	MH536474	MH550794
P79bC	*H13*	MH536460	MH536502	MH550796
P79bD	*H13*	MH536461	MH536502	MH550710
P79bE	*H8*	MH536418	MH536494	MH550776
P79bF	*H1*	MH536396	MH536477	MH550711
P86A	*H25*	MH536419	MH536476	MH550712
P86B	*H15*	MH536431	MH536473	MH550713
P86C	*H26*	MH536397	MH536482	MH550777
P86D	*H11*	MH536398	MH536482	MH550714
P86E	*H1*	MH536399	MH536489	MH550778
PDL1	*H14*	MH536472	MH536502	MH550715
PDL2	*H13*	MH536462	MH536502	MH550792
PDL3	*H1*	MH536400	MH536480	MH550779
PDL5	*H1*	MH536401	MH536478	MH550733
PDL6	*H1*	MH536402	MH536497	MH550793
PNH1	*H5*	MH536445	MH536473	MH550780
PNH2	*H16*	MH536352	MH536490	MH550716
PNH3	*H1*	MH536403	MH536474	MH550717
PNH4	*H5*	MH536416		
PNH5	*H1*	MH536404	MH536477	MH550718
PNH6	*H15*	MH536432	MH536473	MH550734
SAR1	*H15*	MH536433	MH536473	MH550719
SAR2	*H15*	MH536436	MH536473	MH550781
SAR3	*H7*	MH536435	MH536473	MH550720
SAR4	*H13*	MH536463	MH536502	MH550721
SAR5	*H15*	MH536434	MH536473	MH550782
SRB3	*H1*	MH536405	MH536474	MH550783
SRB5	*H13*	MH536464	MH536502	MH550722
SRB6	*H15*	MH536437	MH536473	MH550784
SSR2	*H1*	MH536406	MH536477	MH550785
SSR3	*H1*	MH536407	MH536482	MH550723
SSR4	*H13*	MH536465	MH536505	MH550724
SSR5	*H13*	MH536466	MH536505	MH550725
SSR6	*H15*	MH536438	MH536473	MH550726
TMC1	*H13*	MH536467	MH536502	MH550727
TMC5	*H13*	MH536468	MH536502	MH550728
TMC6	*H13*	MH536469	MH536503	MH550729
VTM1	*H15*	MH536439	MH536473	MH550730

Code	*	COI	nad5	RpS5
VTM2	*H13*	MH536471	MH536502	MH550731
VTM3	*H15*	MH536441	MH536473	MH550788
VTM4	*H15*	MH536440	MH536473	MH550795
VTM5	*H13*	MH536470	MH536503	MH550789
VTM6	*H1*	MH536408	MH536474	MH550790

* Fig 2 (main text) haplotypes
