# Supplementary material for: Uncovered variability in olive moth (Prays oleae) questions species monophyly
Source: PLoS One. 2018 Nov 26;13(11):e0207716. doi: 10.1371/journal.pone.0207716 (PMC6261264; doi:10.1371/journal.pone.0207716)
Supplement: S3 Table — (PDF) [file pone.0207716.s006.pdf]

Supplemental information

Uncovered variability in olive moth (*Prays oleae*) questions species monophyly

Tânia Nobre[(1)]*, Luis Gomes[(1)], Fernando Trindade Rei[(1)]
[(1)] Laboratory of Entomology, ICAAM, University of Évora
* Corresponding author: tnobre@uevora.pt

S3 Table: Variables analysed, including bioclimatic and land cover derived maps

Cod.	Description of variables	Retained*	Source
**DisOG**	Distance to homogeneous olive groves (> 1 ha)	x	[1]
**DenOG**	Density of olive groves per km^2	x	[1]
**DisRG**	Distance to riparian gallery	x	[1]
**Tmax**	mean temperature of the warmest month of the year	x	[2]
**Tmin**	mean temperature of the coldest month of the year	x	[2]
**Tp**	Annual positive temperature	x	[2]
**Pp**	Positive precipitation		[2]
**M**	mean maximum temperature of coldest month	x	[2]
**m**	mean minimum temperature of the coldest month	x	[2]
**Ic**	Simple continentality index	x	[2]
**It**	Thermicity index	x	[2]
**Itc**	Compensated thermicity index	x	[2]
**Ios4**	Ombrothermic index of the summer quarter plus the previous month		[2]
**Ios2**	Ombrothermic index of the warmest bimonth of the summer quarter		[2]
**Ios3**	Ombrothermic index of the summer quarter		[2]

Cod.	Description of variables	Retained*	Source
**Io**	Annual ombrothermic index		[2]
**Mac05**	Macrobioclimates (Rivas-Martínez 2005)	x	[2]
**Mac07**	Macrobioclimates (Rivas-Martínez 2007)		[2]
**Mac0811**	Macrobioclimates (Rivas-Martínez 2008,2011)		[2]
**MB05**	Macrobioclimates and bioclimatic variants (Rivas-Martínez 2005)		[2]
**MB07**	Macrobioclimates and bioclimatic variants (Rivas-Martínez 2007)		[2]
**MB00811**	Macrobioclimates and bioclimatic variants (Rivas-Martínez 2008, 2011)		[2]
**Bio05**	Bioclimates (Rivas-Martínez 2005)		[2]
**Bio07**	Bioclimates (Rivas-Martínez 2007)		[2]
**Bio0811**	Bioclimates (Rivas-Martínez 2008, 2011)		[2]
**Mac05C**	Macrobioclimates (highlighting vernal-aestival indices compensation) (Rivas-Martínez 2005)		[2]
**Mac07C**	Macrobioclimates (highlighting vernal-aestival indices compensation) (Rivas-Martínez 2007)		[2]
**Mac0811C**	Macrobioclimates (highlighting vernal-aestival indices compensation) (Rivas-Martínez 2008, 2011)	x	[2]
**Mac05Cc**	Macrobioclimates (highlighting vernal-aestival indices compensation considering the annual ombrothermic index) (Rivas-Martínez 2005)		[2]
**Mac07Cc**	Macrobioclimates (highlighting vernal-aestival indices compensation considering the annual ombrothermic index) (Rivas-Martínez 2007)		[2]
**Mac0811Cc**	Macrobioclimates (highlighting vernal-aestival indices compensation considering the annual ombrothermic index) (Rivas-Martínez 2007, 2011)		[2]
**Ic05**	Simple continentality (Rivas-Martínez 2005)		[2]
**Ic070811**	Simple continentality (Rivas-Martínez 2007, 2008, 2011)	x	[2]
**Ther05**	Thermotypes (Rivas-Martínez 2005)		[2]
**Ther07**	Thermotypes (Rivas-Martínez 2007)		[2]

Cod.	Description of variables	Retained*	Source
**Ther0811**	Thermotypes (Rivas-Martínez 2008, 2011)	x	[2]
**Omb**	Ombrotypes		[2]
**PpDY**	Positive precipitation for dry year – it corresponds to precipitation for dry year of Nicolau (2002)		[2]
**PpHY**	Positive precipitation for humid year – it corresponds to precipitation for humid year of Nicolau (2002)	x	[2]
**EqOtDY**	Equivalent ombrothermic index for dry year		[2]
**EqOtHY**	Equivalent ombrothermic index for humid year		[2]
**EqOtypeDY**	Equivalent ombrotypes for dry year		[2]
**EqOtypeHY**	Equivalent ombrotypes for humid year		[2]
**OAnoDY**	Ombrothermic index anomaly for dry year		[2]
**OAnoHY**	Ombrothermic index anomaly for humid year		[2]

* X indicate variables retained after covariate correlation check

[1] Obtained by GIS analysis based on Portuguese landcover cartography (IGEO, 2016)

[2] Monteiro-Henriques T, Martins MJ, Cerdeira JO, Silva P, Arsénio P, Silva, et al. Bioclimatological mapping tackling uncertainty propagation: Application to mainland Portugal. Int J Climatol. 2016;36: 400–411. doi:10.1002/joc.4357
